# Cervical Power Doppler Angiography with Micro Vessel Blood Flow Indices in the Auxiliary Diagnosis of Acute Cervicitis

**DOI:** 10.3390/diagnostics12051131

**Published:** 2022-05-03

**Authors:** Yi-Cheng Wu, Ching-Hsuan Chen, Yi-Li Ko, Jack Yu-Jen Huang, Chiou-Chung Yuan, Peng-Hui Wang, Ching-Hua Hsiao, Woei-Chyn Chu

**Affiliations:** 1 Department of Biomedical Engineering, National Yangming Chiaotung University, Taipei 112304, Taiwan; yicheng97.y@nycu.edu.tw; 2 Department of Obstetrics and Gynecology, Taiwan IVF Group, Ton-Yen General Hospital, Zhubei 302048, Taiwan; jyjhuang@stanford.edu; 3 Department of Gynecology, Koo Foundation SYS Cancer Center, Taipei 112019, Taiwan; 4 Department of Obstetrics and Gynecology, Fuyou Branch, Taipei City Hospital, Taipei 100027, Taiwan; chchen@tpech.gov.tw; 5 Nursing Department, Fu Jen Catholic University, New Taipei 242062, Taiwan; 071462@mail.fju.edu.tw; 6 Department of Obstetrics and Gynecology, Cheng Hsin General Hospital, Taipei 112401, Taiwan; ccyuan62@gmail.com; 7 Department of Obstetrics and Gynecology, Taipei Veterans General Hospital, Taipei 112201, Taiwan; phwang@vghtpe.gov.tw; 8 Institute of Clinical Medicine, National Yang-Ming Chiao-Tung University, Taipei 112304, Taiwan

**Keywords:** power Doppler ultrasound, acute cervicitis, pelvic inflammation disease

## Abstract

We have conducted cervical imaging of uterine and micro-vessel flow velocity waveforms in acute pelvic inflammatory disease (PID) by transvaginal power Doppler ultrasound (TVPDU) in order to explore the associations of sonographic parameters with simple and complex cervicitis. Thirty-eight patients with acute PID (26 with acute simple cervicitis and 12 with complex cervicitis) were enrolled for an assessment of vascular grading of cervix and micro-vessel flow velocity using TVPDU before treatment. Seven parameters, including vascular grading (VG), lowest pulsatility index (PI), resistance index (RI), peak systolic velocity (PS), end diastolic velocity (ED), time average maximum velocity (TAMV), and vascular index (VI = PS/ED), were measured and recorded. Forty-one healthy patients were assessed as the control group. Vascular grading (VG) was significantly higher in the study group than the control group (*p* < 0.0001). The PI, RI, and VI were significantly lower in the study group than control group (*p* < 0.0001). No significant associations were observed between seven sonographic parameters and acute simple or/and complex cervicitis. For acute simple cervicitis, a PI cutoff of 1.1 had a sensitivity of 85.4% and a specificity of 92.1% (area under ROC curve [AUC], 93.2%). A RI of 0.6 had a sensitivity of 85.4% and a specificity of 78.9% (AUC, 86.1%). A VI of 2.6 had a sensitivity of 85.4% and a specificity of 78.9% (AUC, 84.9%). Power Doppler angiography of micro-vessel flow velocity waveforms in the cervix could represent a practical method to assist the diagnosis of pelvic inflammatory disease presented as acute cervicitis detected on transvaginal ultrasound before medical or surgical treatment. Cervical PI may be a useful index to detect micro-vessel flow velocity waveforms in acute cervicitis and differentiate acute simple cervicitis from complex cervicitis.

## 1. Introduction

Pelvic inflammatory disease (PID) is a term commonly used to describe inflammation resulting from an infection in the upper genital tract that is not associated with pregnancy or intraperitoneal surgery. Acute PID is caused by an infection ascending along the mucosal surface, which the bacteria colonize. Traditionally, acute PID is assessed quantitatively by measuring the blood flow indices of ovarian, tubal, adnexal masses or uterine vessels using transabdominal or transvaginal color Doppler ultrasound [1,2,3]. Transabdominal ultrasound provides a global overview of pelvic structure and can detect large pelvic masses (such as tubo-ovarian abscesses). Transvaginal sonography can clearly demonstrate and accurately detect inflammation of cervix that may be missed by transabdominal sonography. Moreover, in early acute PID, power Doppler ultrasound has the advantages of high sensitivity for detecting weak blood flow velocity, direction independence, and provides good edge definition of the lumen of the vessels [4,5]. Transvaginal power Doppler ultrasound can also examine the morphology of micro-vessels in more detail and be used to directly assess the microcirculation of the cervix [5]. Many studies have performed hemodynamic assessments in PID by transvaginal color Doppler ultrasound [1,2,3,6]. However, few studies have investigated the hemodynamics of the cervix [1,4].

The main aim of this study was to assess the detailed morphology and blood flow velocity of micro-vessels in the cervix by using transvaginal power Doppler ultrasound in patients with PID who presented with acute cervicitis. Moreover, we examined the value of these parameters to differentiate simple cervicitis from complex cervicitis.

## 2. Materials and Methods

### 2.1. Study Design and Patient Population

Fifty female patients with clinically suspected pelvic inflammatory disease (PID) who presented as acute simple cervicitis or complex cervicitis were recruited at Taipei Veteran General Hospital from January 2000 to August 2021. The inclusion criteria were the typical clinically suspected signs of pelvic inflammatory disease, such as the presence of both positive lifting pain and motion tenderness of the cervix on pelvic examination before clinical treatment. Seven cases without these clinical typical signs, three cases without lifting pain and motion tenderness of the cervix, two cases who received NSAID pain relief before arriving at hospital were excluded from this study. A total of 38 cases met the inclusion criteria; the general characteristics of these patients are presented in Table 1. Forty-one patients with a normal, pathologically proven healthy cervix were recruited as the control group.

Among our case group, 26 patients with acute simple cervicitis were categorized into group 1, and 12 patients with acute complex cervicitis into group 2 (Table 1).

### 2.2. Transvaginal Ultrasound with Power Doppler Angiography (TV-PDA)

All women were scanned using color Doppler ultrasound (Diasonics, Gateway, ST Louis, MO, USA) equipped for color Doppler imaging and color Doppler angiography in the semi-lithotomy position with an empty urinary bladder within one day before undergoing treatment. A 7.0-MHz curved-array endo-vaginal probe was used; the field of view was set to 112 degrees. The uterine cervix was scanned and identified carefully in longitudinal view in grayscale mode. After shifting to angio mode, the color gain was adjusted from 105 to 115, the scale was adjusted to pulse repetition frequency (PRF) and set at 800–900 Hz to filter out low-strength signals, and the temporal filter was adjusted to 1.0 to exclude the blue color within the region of interest (box). Vascularity within the uterine cervix was detected in axial view.

We classified vascularity within the uterine cervix into five grades under power mode color Doppler ultrasound (Table 2), as follows:

### 2.3. Measurement of Blood Flow Velocity Waveforms within the Uterine Cervix

After visually classifying the grade of vascularity from the power mode color Doppler ultrasound, we activated pulse Doppler mode and adjusted the Doppler gate over the target color area to set the gate distance appropriately and adjust the angle of the sampling site to less than 65 degrees. We defined detectable Doppler signals as a series of reproducible, similar arterial waveforms obtained for at least three separate, consecutive cardiac cycles.

Satisfactory Doppler signals were processed and analyzed using online spectra. All measurements were calculated manually, including peak systolic velocity (PS), end diastolic velocity (ED), time-averaged maximum velocity (TAMVV), the pulsatility index (PI), the resistance index (RI), and the vascular index (VI). If more than one set of satisfactory samples were achieved, the lowest set of PI, RI and VI values were recorded. The pulsatility index was defined as the ratio of the difference between the peak systolic and end diastolic velocity to mean velocity. The resistance index was defined as the ratio of the difference between the peak systolic velocity and end diastolic velocity to peak systolic velocity. The vascular index was defined as the peak systolic velocity divided by end diastolic velocity (PS/ED). All scans were performed and assessed by one author (Y.C.W.) to avoid interobserver and intra-observer variation. This study was approved by the Hospital’s Internal Review Board with IRB No.108062.

### 2.4. Statistical Analysis

The vascular grading between study groups and cervical micro-vessel flow waveform parameters were analyzed with Mann-Whitney U test or Kruskal-Wallis test. Post hoc analysis was conducted with and Benjamin-Hochberg procedure. Relationship be-tween vascular grading and waveform parameters were accessed with Spearman’s rank correlation. Clopper-Pearson exact method was used to estimate the testing accuracy confidence intervals. The Statistical Package for the Social Sciences (SPSS) for Windows, version 22.0 (SPSS Inc., Chicago, IL, USA) was used for data analysis and ROC curve analysis. *p*-values < 0.05 were considered statistically significant.

## 3. Results

The mean age of all 79 individuals included in this study was 34.5 (range, 18–51-years-old); all individuals were pre-menopausal. The mean ages of the case group (33.3 ± 7.4) and control group (35.6 ± 6.1) were not significantly different (*p* = 0.159).

In the case group, 26 patients (26/38, 68.4%) had acute simple cervicitis and 12 patients (12/38, 31.6%) had acute complex cervicitis. Most cases of acute complex cervicitis were acute cervicitis complicated by tubo-ovarian abscesses (5/12, 41.7%).

### 3.1. Visual Grading of Vascularity Findings on TV-PDU

The pick-up rate was 100% (38/38) for the case group and 26.8% (11/41) for the control group. The absence of vascularity was classified as grade 0. In the control group, 73.2%, 24.4%, and 2.4% of the individuals were classified as grade 0, grade 1, and grade 2, respectively.

**Figure 1:**
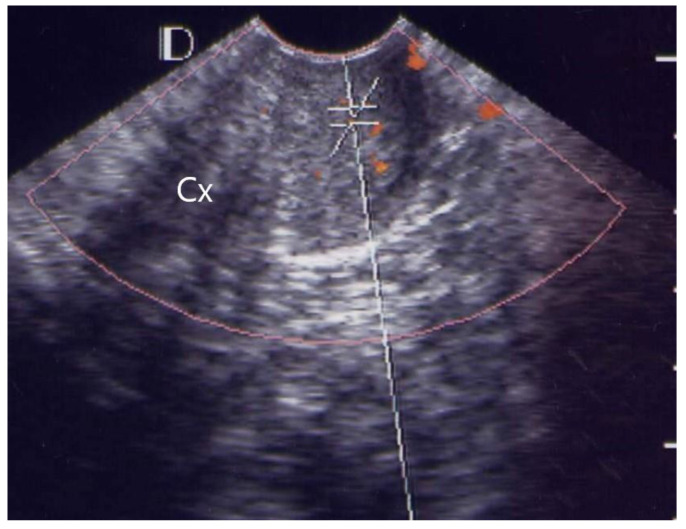
GRADE 1. A case of 46 years old female with pelvic inflammatory disease, less than 5 vascular spots (orange color) in the image of cervix. (Cx: Cervix).

**Figure 2:**
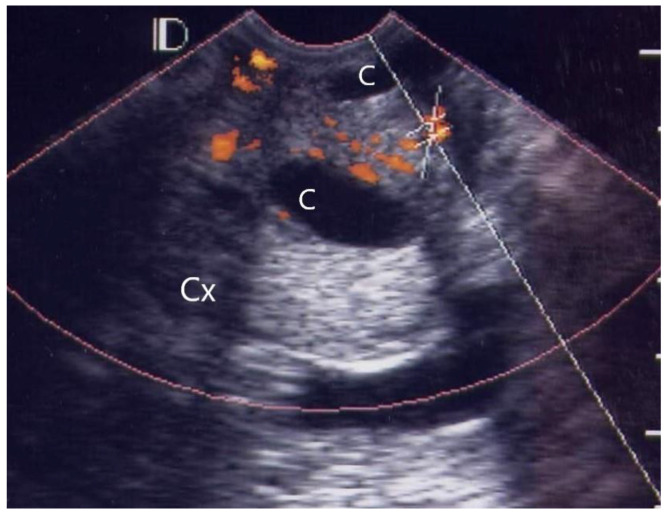
GRADE 2. A case of 29 years old female with pelvic inflammatory disease and tubo-ovarian abscess, more than 5 vascular spots (orange color) are located between two small cervical cysts, but not involve the endocervical canal. (C: Nabothian cyst, Cx: Cervix).

**Figure 3:**
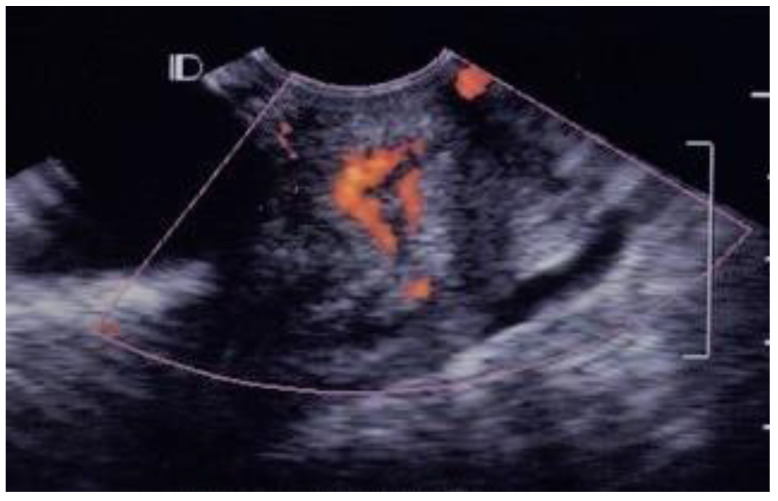
GRADE 3. A case of 22 years old young lady with pelvic inflammatory disease and right ovarian cyst, many vascular spots involved the endocervical canal, without involved whole endocervix.

**Figure 4:**
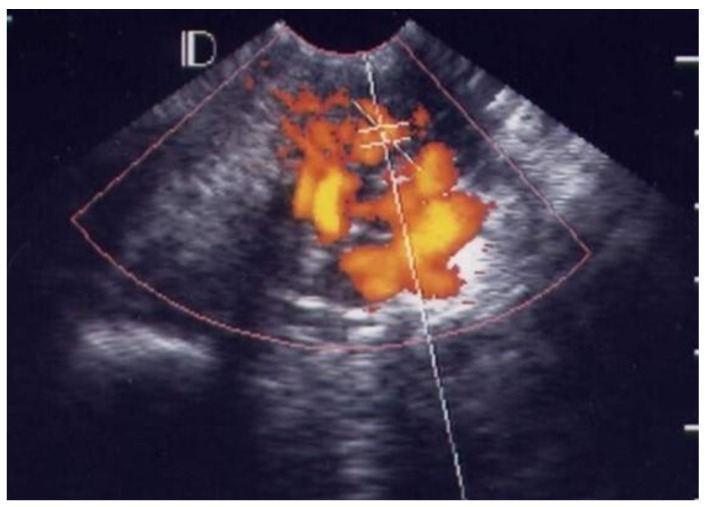
GRADE 4. A case of 18 years old young lady with pelvic inflammatory disease, many vascular spots involved the endocervical canal and whole endocervix.

Of the 38 patients with acute pelvic inflammatory disease, 25 (65.8%) cases were classified as grade 1 (Figure 1), ten (26.3%) as grade 2 (Figure 2), two (5.3%) as grade 3 (Figure 3) and one (2.6%) as grade 4 (Figure 4, Table 1).

In the visible grading of vascularity for group 1 (acute simple cervicitis), 19 cases (19/26, 73.1%) were classified as grade 1, five (5/26, 19.2%) as grade 2, one (1/26, 3.9%) as grade 3, and one (1/26, 3.9%) as grade 4. In group 2, four patients (4/12, 33.3%) were classified as grade 1, seven (7/12, 58.3%) as grade 2, and one patient (1/12, 8.3%) as grade 3.

The vascular grade has significant difference between acute cervicitis and control group, acute cervicitis group has higher grade than control group (*p* < 0.000). In three group comparison, Mann Whitney U test with multiple comparison adjustment showed significant differences between cervicitis and control group (complex cervicitis group > normal, *p* < 0.000; simple cervicitis group > normal, *p* < 0.000). The difference be-tween complex and simple cervicitis was insignificant (*p* = 0.076).

**Figure 5:**
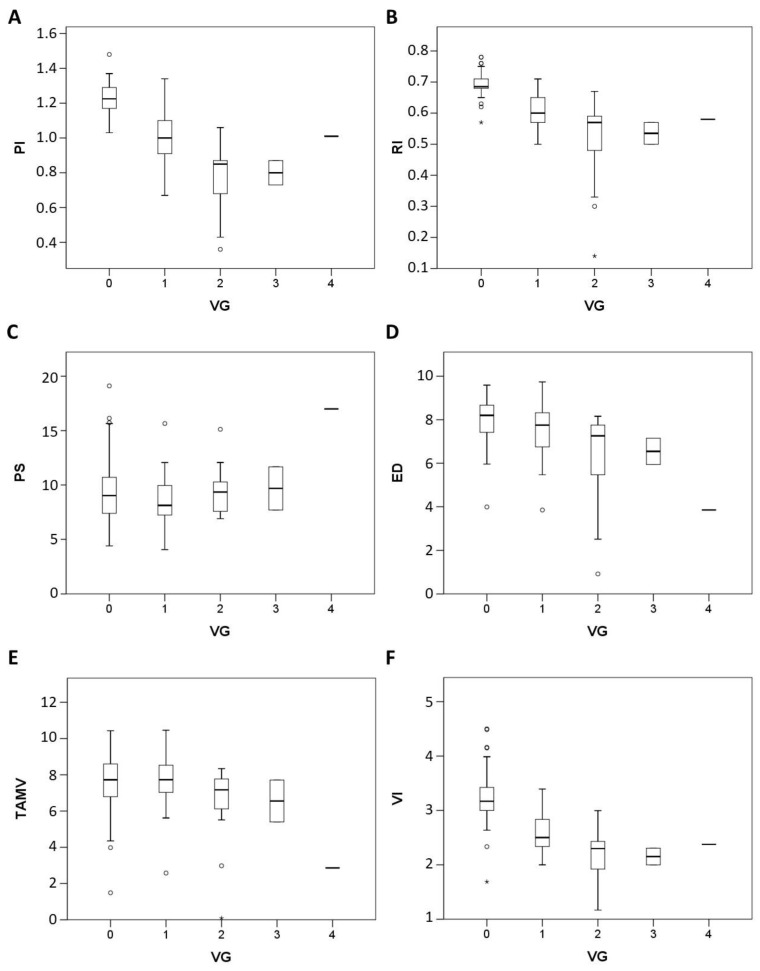
Box plot showing the correlation between the vascular grading (VG) and 6 sonographic parameters of cervix (PI, RI, PS, ED, TAMV, VI) in study group (N = 38) and control group (N = 41). (**A**,**B**,**D**,**F**): A negative correlation can be seen in PI, RI, ED and VI. (**C**,**E**): No specific trend in PS and TAMV. * Extreme outlier, o Outlier.

The vascular grade is negatively correlated with PI (−0.8, *p* = 0.000), RI (−0.7, *p* = 0.000), VI (S/D) (−0.7, *p* = 0.000), ED (−0.4, *p* = 0.000), but there is no correlation with PS (−0.02, *p* = 0.858) and TAMV (−0.2, *p* = 0.068) (Spearman rho correlation coefficient analysis) (Figure 5).

### 3.2. Micro-vessel Flow Velocity Waveform Findings on TV-PDU

The mean PI was significantly different between the study group (0.9 ± 0.2) and control group (1.2 ± 0.1; *p* = 0.0001). Similarly, the mean RI was also significantly different between the study group (0.6± 0.1) and control group (0. 7 ± 0.1; *p* = 0.0001). However, the mean VI was significantly different between the study group (2.4 ± 0.5) and control group (3.1 ± 0.6; *p* = 0.0001).

**Figure 6:**
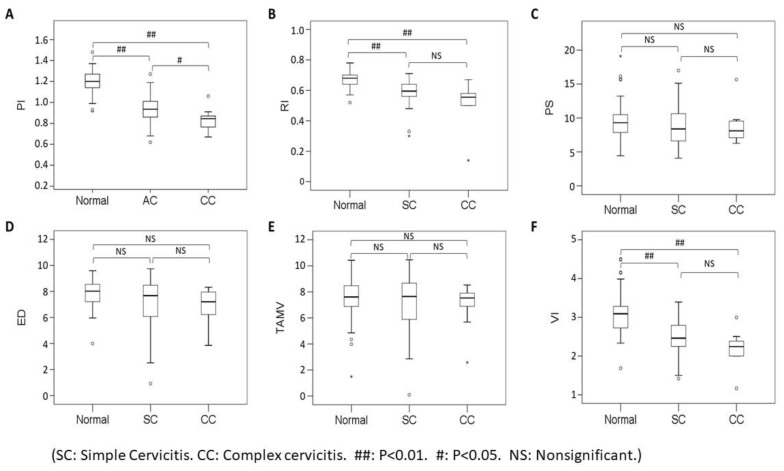
Box plot showing the correlation between 6 sonographic parameters of cervix (PI, RI, PS, ED, TAMV, VI) among the simple cervicitis group (SC) (*n* = 26), complex cervicitis group (CC) (*n* = 12) and control group (*n* = 41). (**A**) Disease severity is negatively correlated with PI. (**B**,**F**) RI and VI are lower in the cervicitis groups. (**C**,**D**,**E**) Patients with cervicitis and the control groups have similar distributions of PS, ED and TAMV. * Extreme outlier, o Outlier.

PI, RI, and VI differ significantly in the comparison of complex cervicitis vs. simple cervicitis vs. control group. (*p* < 0.05). PI, RI, and VI differ significantly in the comparison of cervicitis and control group. (*p* < 0.05). PI differ significantly in the comparison of complex cervicitis vs. simple cervicitis. (*p* < 0.05) (Mann Whitney U test and Kruskal Wallis test) (Figure 6).

**Figure 7:**
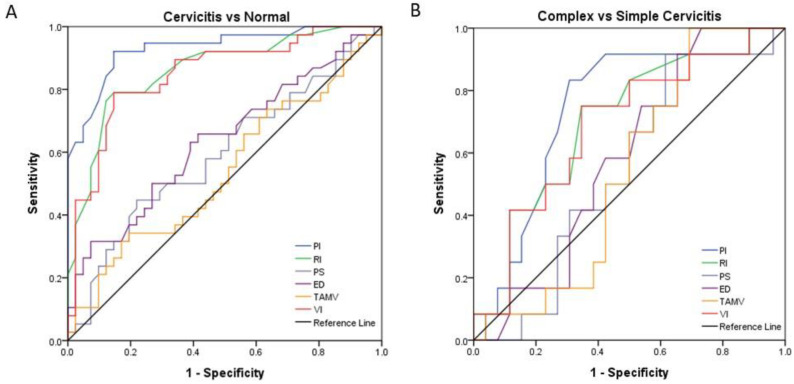
(**A**) ROC curve of six sonographic parameters between the case group (*n* = 38) and control group (*n* = 41). (**B**) ROC curve of six sonographic parameters between the group 2 (*n* = 12) and group 1 (*n* = 26).

A PI cutoff value of 1.1 had a sensitivity of 85.4%, specificity of 92.1%, positive predictive value (PPV) of 91.5%, negative predictive value (NPV) of 86.3%, and accuracy of 93.2% for acute simple cervicitis. A RI cutoff value of 0.6 had a sensitivity of 85.4%, specificity of 78.9%, PPV of 80.2%, NPV of 84.4%, and accuracy of 86.1% for acute simple cervicitis. A VI cutoff value of 2.6 had a sensitivity of 85.4%, specificity of 78.9%, and accuracy of 84.9% for acute simple cervicitis (Figure 7A, Table 3).

B. ROC curve of six sonographic parameters between the group 2 (*n* = 12) and group 1 (*n* = 26).

The PI of the cervix was the best sonographic index for detection of micro-vessel flow velocity waveforms in acute simple cervicitis. Moreover, the PI of the cervix was also the best index to differentiate between acute simple cervicitis and complex cervicitis; a PI cutoff value of 0.9 had a sensitivity of 69.2%, specificity of 83.3%, PPV of 80.6%, NPV of 73.0%, and accuracy of 74.2% for discriminating between complex cervicitis and simple cervicitis (Figure 7B, Table 3).

## 4. Discussion

Tissue inflammation is accompanied by increased blood flow, dilatation of vessels, and angiogenesis. Pelvic inflammatory disease (PID) is difficult to diagnose, as most of the symptoms are often mild and subtle [6]. Transvaginal ultrasound (TVS) is a useful technique for the diagnosis of pelvic inflammatory disease (PID), especially when combined with power Doppler mode to detect the blood flow in micro-vessels with slow velocity [5,7,8]. Detection of inflammation-induced hyperemia by power Doppler ultrasound in PID is feasible, as further shown in this study. However, most studies of acute PID focused on acute salpingitis, endometritis or other unknown etiologies [9,10,11]. Moreover, spectral Doppler studies usually measure the bilateral uterine artery, tubal artery, or ovary artery [2,10,11]. In the acute phase of pelvic infection, vascular resistance (pulsatility index, resistance index) is low in the uterine arteries, tubo-uterine and ovarian arteries and arteries of pelvic masses and returns to normal when the infection subsides [2,12,13].

In acute PID presenting with simple cervicitis, most vascular hotspots were classified as grade 1 and grade 2 (24/26, 92.0%) using our visual grading system. Moreover, most cases of complex cervicitis were classified as grade 1 and grade 2 (11/12, 91.7%) on the visual grading system. That is, if the grading system is greater than or equal to 1, it is regarded as acute cervicitis, the sensitivity can reach 100%, and the specificity can reach 73.1% (Table 1). These results reflect the clinical symptoms of acute PID, as most symptoms are mild and subtle. However, this vascular hotspot grading system of micro-vessel flow velocity waveforms using TV-PDU represents a simple, no time-consuming feasible measurement tool compared to 3D ultrasound [14,15,16,17,18,19], which requires complete reconstructions.

In normal healthy cervical tissue, color angio mode 3D ultrasound detects some small vessels in the myometrium, but no other color-coded areas in the submucosal layer, similarly to our observations for grade 0, grade 1, or grade 2. In our control group, 30/41 cases were grade 0, 10/41 were grade 1 and only one case was grade 2. Inflammation due to bacterial or viral cervicitis leads to angiogenesis. A previous study using 3D ultrasound in color angio mode revealed the arterial blood supply of the submucosal layer spreading into an arterial spiral of vessels running down to the portion of cervix, which is compatible with our observations of grade 3 and grade 4 vascularity within the cervix [14]. The 3D color Power angio mode image of cervical vessels in a patient with bacterial cervicitis corresponds to our grade 3. The result shown in Figure 3 of the previous study is similar to the grade 4 of our visual grading.

The main sonographic parameters ever applied in patients with acute PID, including the pulsatility index (PI), peak systolic velocity (PSV) and time-averaged maximum velocity (TAMV) [9,10]. Molander et al. [9] reported all have hyperemia in patients with acute PID (*n* = 20) and a significantly lower PI (0.84 ± 0.04) in the blood vessels of the tubal walls and adnexal masses of women with acute PID than a control group (1.5 ± 0.1) (*p* < 0.01). Even in different blood vessels, in our study, we also have the similar results about the pick-up rate was 100% (38/38) and a significant lowest mean PI (0.9 ± 0.2) in our case group. Romosan et al. [10] assessed the PSV, TAMV and PI of the bilateral uterine artery and tubal artery in severe salpingitis (*n* = 17), endometriosis (*n* = 9), cervicitis (*n* = 3) and unrelated to genital infection (*n* = 23). A review of the literature from Romosan et al. [11]. showed that the spectral Doppler results for women with and without acute PID overlap too much to enable the diagnosis of acute PID; but they found lower vascular resistance and higher blood flow velocities in salpingitis compared with the other groups. Even so, transvaginal ultrasound is likely to be helpful when managing woman with symptoms of acute PID.

In our study, we also have two main findings, one of which is the vascular grade is negatively correlated with PI (−0.8, *p* = 0.000), RI (−0.7, *p* = 0.000), VI (−0.7, *p* = 0.000) (Figure 5). It is reasonable that infected tissue is prone to induce angiogenesis, so the lower index value from spectral Doppler analysis, the higher vascular grading within cervix was demonstrated from this study. The second is even the PI, RI and VI(S/D) index value decrease with the severity of cervicitis. (*p* < 0.05), but PI is best than RI and VI because it has significant difference between simple cervicitis and complex cervicitis (*p* < 0.05) (Figure 6). Although we know that complex cervicitis is more severe than simple cervicitis, because the number of cases in this study is limited, it is unable to know whether the two diseases occur sequentially or simultaneously.

Moreover, gently, and slightly touching, no need to apply the same pressure on the vaginal probe every time [20], this micro-vessel flow velocity waveforms could supply full quantitative analysis of cervix tissue in benign disease of cervix [21,22], especially in acute simple cervicitis and complex cervicitis.

## 5. Conclusions

Vascular grading system using by transvaginal power Doppler angiography can help us quickly diagnose if there is acute hyperemia of cervix. For acute simple cervicitis, a PI cutoff of 1.1 had a sensitivity of 85.4% and specificity of 92.1% (area under ROC curve [AUC], 93.2%). Cervical PI may be a useful index to detect micro vessel flow velocity waveforms in acute cervicitis and differentiate acute simple cervicitis from complex cervicitis. Our study could provide a practical method to assist diagnosing of acute cervicitis detected on transvaginal ultrasound before medical or surgical treatment.

## Figures and Tables

**Table 1:** The characteristics of case group and control group in Vascular Hot Spot Grading System by using TV-PDU.

	Number	Vascular Hot Spot Grading				
		Grade 0	Grade 1	Grade 2	Grade 3	Grade 4
**Simple Cervicitis**	26	0	19	5	1	1
**Complex Cervicitis**	12	0	6	5	1	0
Cervicitis + TOA	5	0	1	4	0	0
Cervicitis + PCS	2	0	1	1	0	0
Cervicitis + PCO	1	0	1	0	0	0
Cervicitis + RO	1	0	1	0	0	0
Cervicitis + BC	1	0	1	0	0	0
Cervicitis + ROC	1	0	0	0	1	0
Cervicitis + TOA+ OC	1	0	1	0	0	0
Normal Cervix	41	30	10	1	0	0

TV-PDU: Transvaginal Power Doppler Ultrasound, TOA: Tubo-ovarian abscess, PCS: pelvic congestion syndrome, PCO: polycystic ovary, RO: Right oophoritis, BC: Batholin’s cyst, ROC: Right ovarian cyst, OC: Ovarian cyst.

**Table 2:** Visual vascular hotspot grading system.

Grading	Numbers of Vascular Hot Spot within Cervix (One Spot: 1 × 1 mm)
Grade 0	Absence of vascular hot spot
Grade 1	<5 vascular spots, not involve the endocervical canal
Grade 2	>5 vascular spots, not involve the endocervical canal
Grade 3	Involved the endocervical canal, without involved whole endocervix
Grade 4	Involved the whole endocervix

**Table 3:** Optimal cutoff values of six sonographic parameters for discriminating between the case group (*n* = 38) and control group (*n* = 41). It is also between complex cervicitis group (N = 12) with simple cervicitis group (N = 26).

**A: Optimal Cutoff of Six Sonographic Parameters for Discriminating Acute Cervicitis Group (N = 38) with** **Control Group (N = 41)**								
**Area under the Curve**								
**Variables**	**Area**	**Optimal**	**Sensitivity**	**95%LCL**	**95%UCL**	**Specificity**	**95%LCL**	**95%UCL**
PI	0.93	1.1	92.1	78.6	98.3	85.4	70.8	94.4
RI	0.86	0.6	78.9	62.7	90.4	85.4	70.8	94.4
PS	0.59	8.5	50	33.3	66.6	56.1	39.7	71.5
ED	0.64	3.1	65.8	48.6	80.4	58.5	42.1	73.7
TAMV	0.54	5.2	60.5	43.4	75.9	46.3	30.7	62.6
VI	0.85	2.6	78.9	62.7	90.4	85.4	70.8	94.4
**B: Optimal Cutoff of Six Sonographic Parameters for Discriminating Complex Cervicitis Group (N = 12) with Simple Cervicitis Group (N = 26)**								
**Area under the Curve**								
**Variables**	**Area**	**Optimal**	**Sensitivity**	**95%LCL**	**95%UCL**	**Specificity**	**95%LCL**	**95%UCL**
PI	0.74	0.9	83.3	51.6	97.9	69.2	48.2	85.7
RI	0.69	0.6	75	42.8	94.5	65.4	44.3	82.8
PS	0.55	8.6	66.7	34.9	90	50	29.9	70.1
ED	0.58	3.3	75	42.8	94.5	46.2	26.6	66.6
TAMV	0.54	5.4	50	21.1	78.9	50	29.9	70.1
VI	0.69	2.4	75	42.8	94.5	65.4	44.3	82.8

VG: Vascular grading, PI: Pulsatility index, RI: Resistance index, PS: Peak systolic velocity, ED: Endiastolic velocity, TAMV: Time average maximum velocity, VI: Vascular index, LCL: Lower confidence limit, UCL: Upper confidence limit.

## Data Availability

The Excel data used to support the findings of this study were supplied by Yi-Cheng Wu under license, and requests for access to these data should be made to Yi-Cheng Wu, wu102007@gmail.com.
